# Bone Mineral Accrual From Adolescence Into Young Adulthood and Peak Bone Mass: A Longitudinal Cohort Study

**DOI:** 10.1002/hsr2.71411

**Published:** 2025-10-28

**Authors:** Arzhang Naseri, Farnaz Atighi, Alireza Keshtkar, Pedram Talezadeh, Marzieh Bakhshayeshkaram, Marjan Jeddi, Mohammad Mahdi Dabbaghmanesh, Seyed Taghi Heydari, Naeimehossadat Asmarian, Mohammad Hossein Dabbaghmanesh

**Affiliations:** ^1^ Endocrinology and Metabolism Research Center Shiraz University of Medical Sciences Shiraz Iran; ^2^ Research Center for Noncommunicable Diseases Jahrom University of Medical Sciences Jahrom Iran; ^3^ Physiology, Endocrinology and Metabolism Research Center Shiraz University of Medical Sciences Shiraz Iran; ^4^ Internal Medicine, Endocrinologist, Endocrinology and Metabolism Research Center Shiraz University of Medical Sciences Shiraz Iran; ^5^ Biostatistics, Health Policy Research Center Shiraz University of Medical Sciences Shiraz Iran; ^6^ Biostatistics, Anesthesiology and Critical Care Research Center Shiraz University of Medical Sciences Shiraz Iran; ^7^ Internal Medicine, Endocrinologist Shiraz University of Medical Sciences Shiraz Iran

**Keywords:** adolescence, bone mineral apparent density, bone mineral content, bone mineral density, growth, peak bone mass, puberty, young adulthood

## Abstract

**Background and Aims:**

Understanding bone mass accumulation patterns and factors influencing them during growth is important for preventing osteoporosis later in life. This study investigated longitudinal changes in bone mineral content (BMC), bone mineral density (BMD), and bone mineral apparent density (BMAD) during growth, with the aim of estimating the age at which peak bone mass (PBM) is achieved in males and females. It also examined the effects of puberty, age, body mass index (BMI), sex, socioeconomic status (SES), and habitual exercise on these bone parameters during adolescence and early adulthood.

**Methods:**

This retrospective cohort study included 159 participants. Bone densitometry was performed using dual‐energy x‐ray absorptiometry (DXA) during two assessments. Anthropometric measurements were obtained, and demographic data, pubertal stage, medical history, SES, and exercise habits were collected through a structured questionnaire administered by a physician.

**Results:**

Rates of change in total body BMC (TBBMC) and BMD (TBBMD) decreased with advancing age and pubertal stage, with the highest rates observed at ages 9–11 and Tanner stage 1. Across all ages up to 18 years, males showed significantly higher rates than females. Peak TBBMC and TBBMD occurred at 25.7 and 26.2 years in males, and 24.8 and 24.0 years in females. After adjusting for bone size, BMAD peaks at the femoral neck and lumbar spine were 21.2 and 23.8 years in males, and 24.8 and 24.1 years in females. Linear mixed model analysis indicated that older age, higher BMI, male sex, higher SES (significant for BMD), and greater physical activity positively influenced TBBMC and TBBMD until peak values were reached.

**Conclusion:**

Bone accumulation was greater in adolescence than in early adulthood. PBM was generally reached in the mid‐third decade, with females attaining it earlier. Age, sex, BMI, SES, and exercise positively influenced TBBMC and TBBMD during adolescence and early adulthood.

**List of abbreviations**
BA: bone area
BMAD: bone mineral apparent density
BMC: bone mineral content
BMD: bone mineral density
BMI: body mass index
DXA: dual‐energy x‐ray absorptiometry
PBM: peak bone mass
SES: socioeconomic status

## 1 Introduction

Osteoporosis is a critical health concern among the elderly, primarily due to its association with fragility fractures, which significantly increase morbidity, mortality, and healthcare costs. Recent research indicates osteoporosis affects approximately 21.7% of older adults globally [1]. By 2040, it is estimated that the number of people aged 65 and older will double from 2008 figures, constituting about 14% of the global population. Furthermore, the number of individuals aged 80 and above is expected to double from 2010 to 2050 [2]. As the global population ages and developing countries adopt Western lifestyles, the burden of osteoporosis and its related complications is anticipated to escalate markedly worldwide [3].

Osteoporosis is defined by the structural deterioration of bone tissue and a reduction in bone mass. Bone mass plays a significant role in assessing osteoporosis risk and is determined by peak bone mass (PBM) achieved during growth and the subsequent rate of bone loss in later life [4, 5]. PBM refers to the maximum amount of bone tissue that exists at the end of skeletal maturation [6]. Research indicates that higher PBM has a greater impact on bone density than the age‐ or menopause‐associated bone loss that occurs afterward. Notably, up to 60% of the risk of developing osteoporosis can be attributed to the quantity of bone minerals accumulated during adolescence and early adulthood [7]. Therefore, maximizing bone mineral accrual during these years is essential; as a 10% increase in PBM can reduce the risk of osteoporotic fractures in older adults by 50% [8].

The precise age at which PBM is attained is still debatable. There is limited earlier research proposing that bone mass reaches its maximum long after sexual and skeletal maturity, potentially during or later the third decade of life [9, 10]. Conversely, more recent studies suggest that bone mass peaks earlier than previously believed [11, 12, 13]. While several studies have estimated PBM using cross‐sectional data, a limited number have conducted longitudinal assessments, specifically targeting adolescents and young adults. Understanding bone growth patterns in relation to age, sex, and pubertal status along with the age at which PBM is reached, is vital for identifying potential issues in bone mass accumulation during growth. This knowledge can inform interventions before the attainment of PBM. In addition, various factors influence bone mass accumulation, including nonmodifiable factors such as genetics and family history, and environmental and behavioral factors which account for 20%‐40% of the variation [14]. Therefore, promoting behavioral changes during adolescence is essential for developing effective preventive programs aimed at maximizing bone mineral content (BMC) and density (BMD).

This study aimed to examine the longitudinal changes in BMC and BMD at axial sites in males and females from adolescence to early adulthood and estimate the age at which these bone measurements peak. We also sought to relate these changes to factors such as pubertal stage, age, sex, body mass index (BMI), socioeconomic status, and exercise. We utilized dual‐energy X‐ray absorptiometry (DXA) to measure BMC and BMD while also incorporating bone mineral apparent density (BMAD) to address size‐related biases in BMD results.

## 2 Methods

### 2.1 Study Population and Design

This retrospective cohort study was conducted between 2020 and 2023 on individuals who previously participated in the Kawar Children and Adolescents Bone Mineral Density Study in 2010 to 2011 [15].

Those who satisfied the following criteria were included in the study:

‐ Being a resident of Kawar city and not having migrated to other cities or countries for education, work, mandatory military service, or other reasons.

‐ Consenting to participate in the study despite the COVID‐19 outbreak.

‐ Not having a history of precocious or delayed puberty.

‐ Not having systemic diseases such as hyper‐ or hypothyroidism, renal failure, diabetes mellitus, adrenal insufficiency, recurrent fractures, chronic musculoskeletal disorders, or prolonged immobility.

‐ Not taking medications affecting bone metabolism, such as anticonvulsants, glucocorticoids, etc.

‐ Not being pregnant if female.

Two hundred and twenty participants met the inclusion criteria and were enrolled in the study. All participants were evaluated for demographic, clinical, anthropometric, and laboratory evaluations. Among these participants, a total number of 159 individuals agreed to complete the study and underwent further DXA assessments. Participants were divided into four age groups based on their baseline age (i.e., age at the original 2010–2011 assessment): 9–11 years (*n* = 43), 12–14 years (*n* = 42), 15–17 years (*n* = 56), and ≥ 18 years (*n* = 18). The participants who completed the study and the total participants at baseline showed no significant difference in age, sex, BMI, total body BMC and BMD, and other factors associated with bone mass.

All procedures performed in this study involving human participants were in accordance with the ethical standards of The Human Ethics Committee of Shiraz University of Medical Sciences with the approval code of IR.SUMS.REC.1401.351 and with the 1964 Helsinki Declaration and its later amendments or comparable ethical standards. Written informed consent was obtained from all individual participants or their parent/guardian if they were under the age of 18 years before inclusion and after they were fully informed about the study's goals, cooperation, benefits, and potential harms.

### 2.2 Data Collection and Measurements

Demographic information, such as age, sex, marital status, education, employment, and income, lifestyle factors like physical activity, tobacco and alcohol use, and smoking, medical history including current health status, childhood illnesses, chronic illnesses, acute illnesses, past and present medications, surgeries, accidents or injuries, and family history, and reproductive history, such as the age of menarche, gravidity, abortion, lactation, contraception, and the age of menopause, were collected by a physician using a comprehensive, investigator‐designed structured questionnaire—adapted from the Kawar Children and Adolescents Bone Mineral Density Study—that underwent rigorous content validation by a panel of endocrinologists and epidemiologists [15].

The medical doctor followed standardized procedures to measure the weight and height of the individuals [16]. The height was measured at 0.1 cm while the participant stood without shoes, using an electronic portable, wall‐mounted stadiometer (Seca, CA, USA). Weight was measured to 0.1 kg with the participant wearing light indoor clothing without shoes, using a calibrated electronic scale (Seca, CA, USA). BMI (kilograms/square meters) was expressed by dividing weight (kilograms) over height squared (square meters).

Also, according to the International Conference on Consensus Physical Activity Guidelines for Adolescents, which recommends at least 3 days of 20 min or more of moderate‐to‐vigorous activity per week, the participants were asked whether they did this level and duration of physical activity less or more than three times a week [17].

Kuppuswamy's socioeconomic status scale, which is widely used in urban and rural areas to measure socioeconomic status, was used to assess the participants' socioeconomic status. The scale yields a score ranging from 3 to 29, calculated based on three factors: the head of the family's education scored from 1 to 7, the occupation of the head of the family scored from 1 to 10, and the monthly income of the family scored from 1 to 12 [18].

### 2.3 Bone Densitometry

The Hologic Horizon (Hologic Corp, Bedford, MA, USA) was used to measure the bone area (BA, cm^2^), BMC (g), and BMD (g/cm^2^) of the total body, anteroposterior lumbar spine (L1–L4), total hip, and femoral neck by a single qualified technologist according to standard protocols. Participants were dressed in special clothing and no footwear during densitometric studies. The region of interest's position, size, and location were the same for all participants during DXA measurements. A standardized pre‐scan protocol was implemented to reduce biological variability and enhance measurement precision. Participants were instructed to fast overnight (for at least 8 h) and to abstain from moderate‐to‐vigorous physical activity for at least 24 h before the examination to prevent fluid shifts and changes in hydration status. Intake of calcium or other dietary supplements was also prohibited on the morning of the scan [19]. To ensure accurate measurement of the lumbar spine, the participants' knees were elevated in a supine position to eliminate physiological lumbar lordosis and they were instructed to remain completely still and breathe normally to avoid motion artifacts. Before each assessment, the densitometer was calibrated by Phantom. The in vivo coefficient of variation for BMD was 1% for the total body, 0.8% for the lumbar spine, 1.8% for the total hip, and 2.4% for the femoral neck. The BMAD was determined using previously established equations [20]. For the spine, BMAD was calculated according to the following equation: Lumbar Spine BMC/(area)^1.5^, and for the femoral neck it was derived from this one: femoral neck BMC/(area)^2^.

### 2.4 Statistical Analysis

To study the anthropometric characteristics, BMC, and BMD across different age and sex groups, a descriptive analysis was conducted using the Explore method. Mean values and standard deviations (SD) were calculated for each parameter. Data normality was assessed using the Shapiro–Wilk test. Furthermore, we analyzed BMC and BMD patterns at each site, considering participants' Tanner pubertal stages. The Tanner five‐stage puberty classification for each participant was derived from data collected in the previous study, as determined during endocrinologist assessments, based on the development of breasts and pubic hair in girls and genitalia and pubic hair in boys [15, 21].

To define the rate of change for these variables, we calculated the difference between two examinations for each participant and divided this by the time interval between the examinations. Independent samples t‐tests were conducted to compare anthropometric characteristics, BMC, and BMD across age groups by sex, while One‐way ANOVA was used to analyze these variables among different age groups within each sex. Additionally, we calculated the rates of change in BMC and BMD at each site for each Tanner stage group. One‐way ANOVA was also applied to compare baseline values and rates of change in BMC and BMD across various pubertal groups. Following the ANOVA, a post hoc Tukey test was performed to identify specific differences among Tanner stage groups. For all comparisons, mean differences, 95% confidence intervals, and p‐values were calculated to assess the magnitude, precision, and statistical significance of the observed effects, with detailed results of all statistical comparisons, including exact p‐values and post‐hoc tests, provided in Supporting Information S1.

To estimate the age at which PBM is reached, we fitted separate linear regression models for the rates of change in total body BMC and BMD, and lumbar spine and femoral neck BMAD against mid‐point ages. This approach, which models the decline in the rate of bone accrual with age, is well‐established for estimating the age at which the net annual gain approaches zero, indicating a plateau in bone mass. It is important to note that while bone accrual may follow a nonlinear trajectory, this method is designed to estimate the timing of the plateau rather than to model its precise nonlinear shape [9]. As previously mentioned, the rates of change were calculated as the difference between two examinations divided by the time interval between them for each participant. The mid‐point age for each participant was determined by averaging their ages at the first and second measurements. In this analysis, PBM was defined as the age at which the linear regression equation shows a rate of change of zero. This indicates that increases in total body BMC and BMD, and BMAD have stopped beyond this specific age. We conducted all analyzes separately for each sex considering the significant differences in the PBM between males and females.

In addition, we utilized a linear mixed model to explore the impact of various factors—such as age, BMI, sex, socioeconomic status index, and exercise—on total body BMC and BMD, and BMAD at both the lumbar spine and femoral neck. The dependent variables—total body BMC and BMD, and BMAD at lumbar spine and femoral neck—were analyzed individually to identify the specific effects of each predictor on these outcomes. With a total sample size of 159, the study has ≥ 80% power to detect medium effect sizes (d ≈ 0.50; f ≈ 0.25) at α = 0.05, in line with Cohen's guidelines.

The Statistical Package for the Social Sciences version 22.0 (SPSS Inc., Chicago, IL, USA) was used for all statistical analyzes. All tests were two‐sided and a *p*‐value of less than 0.05 was considered statistically significant.

## 3 Result

The study included 159 males (*n* = 81) and females (*n* = 78), with a mean age of 14.14 ± 2.60 and 13.81 ± 3.02 years old, respectively, at their first observation. In the second observation, the mean age was 23.94 ± 2.93 years for males and 23.40 ± 3.28 years for females. The mean duration between the two evaluations was 9.70 ± 1.45 years. Table 1 shows baseline anthropometric characteristics, and BMC and BMD measurements of the lumbar spine, femoral neck, total hip, and total body in all age groups based on sex.

**Table 1:** Baseline anthropometric characteristics, BMC (g), and BMD (g/cm^2^) for each age group according to sex.

	Male (*n* = 81)				Female (*n* = 78)			
	9–11 (*n* = 18)	12–14 (*n* = 23)	15–17 (*n* = 31)	18 ≤ (*n* = 9)	9–11 (*n* = 25)	12–14 (*n* = 19)	15–17 (*n* = 25)	18 ≤ (*n* = 9)
Weight (kg)	30.05 ± 4.75	40.39 ± 11.02	56.74 ± 12.27	60.55 ± 12.13	28.56 ± 5.07	41.73 ± 7.17	50.56 ± 11.09	52.44 ± 9.04
Height (cm)	138.38 ± 6.27	154.69 ± 11.47	169.06 ± 8.30**	176.22 ± 7.79**	137.48 ± 8.71	152.89 ± 5.36	156.36 ± 7.95**	158.88 ± 6.19**
BMI (kg/m^2^)	15.62 ± 1.68	16.62 ± 3.05	19.82 ± 4.08	19.41 ± 3.18	15.04 ± 1.77	17.78 ± 2.45	20.55 ± 3.66	20.75 ± 3.12
Lumbar spine BMC	22.60 ± 3.66	32.01 ± 8.90	50.20 ± 13.16	56.52 ± 13.16	22.68 ± 4.11	35.74 ± 7.10	50.24 ± 9.69	54.35 ± 7.48
Lumbar spine BMD	0.54 ± 0.07	0.67 ± 0.10*	0.82 ± 0.14*	0.90 ± 0.16	0.57 ± 0.06	0.74 ± 0.08*	0.91 ± 0.10*	0.96 ± 0.10
Femur neck BMC	2.93 ± 0.45**	3.39 ± 0.67*	4.38 ± 0.76**	4.71 ± 0.76**	2.48 ± 0.37**	3.01 ± 0.38*	3.55 ± 0.52**	3.75 ± 0.49**
Femoral neck BMD	0.63 ± 0.07*	0.71 ± 0.10*	0.82 ± 0.11	0.84 ± 0.16	0.58 ± 0.06	0.64 ± 0.05*	0.77 ± 0.10	0.78 ± 0.10
Total hip BMC	16.43 ± 2.71	22.91 ± 6.86	33.18 ± 5.91**	34.62 ± 7.64*	15.53 ± 2.84	20.63 ± 3.34	25.20 ± 4.21**	27.68 ± 3.92*
Total hip BMD	0.72 ± 0.08*	0.78 ± 0.10	0.92 ± 0.11	0.92 ± 0.16	0.67 ± 0.06*	0.75 ± 0.07	0.87 ± 0.10	0.90 ± 0.10
Total body BMC	992.55 ± 108.76	1391.11 ± 315.73	1877.35 ± 374.21*	2111.65 ± 455.00	983.66 ± 117.19	1302.92 ± 191.63	1695.21 ± 275.45*	1826.53 ± 242.75
Total body BMD	0.75 ± 0.05	0.86 ± 0.07	0.96 ± 0.09	1.01 ± 0.11	0.74 ± 0.05	0.83 ± 0.06	0.97 ± 0.08	1.00 ± 0.07

*Note:* Statistically significant differences between males and females are given as * for *p* < 0.05 and ** for *p* < 0.01. See Supporting Information S1 for full statistical details (mean differences, 95% CIs, exact *p*‐values).

Abbreviations: BMC, bone mineral content; BMD, bone mineral density; BMI, body mass index.

Our results indicate that height, weight, and axial skeletal sites' BMC and BMD increased significantly in both sexes from age 9 to 18 years. There was no significant difference in height and weight between males and females until age 15, at which point males had their typical pubertal growth spurt marked by significantly higher heights compared to females. BMI did not differ significantly between sexes in all age groups.

As shown in Table 1, although no significant difference existed in lumbar spine BMC measures between males and females in any age group, the lumbar spine BMD was significantly higher in females in age groups 12–14 (*p* < 0.01) and 15–17 (*p* < 0.05). Across all age groups, males had significantly higher BMC in the femoral neck. However, femoral neck BMD was no longer significantly higher in males after 15 years of age. Total hip BMC showed no significant difference between males and females until age 15, where males had significantly higher means. On the other hand, total hip BMD was significantly higher in males than females only in the age group of 9–11, and no significant difference was seen in older ages. There were no significant differences in total body BMC and BMD between males and females, except in 15–17‐year‐old participants, where males had significantly higher TBBMC.

Table 2 presents the rate of changes in anthropometric characteristics, BMC, and BMD measurements across different age groups and sexes. We observed positive changes in weight, height, and BMI for all age groups and both sexes. Similarly, BMC and BMD at the lumbar spine, total hip, and total body increased positively across all ages and in both sexes. However, for males aged 18 years and older, negative changes in BMD and BMC at the femoral neck were observed.

**Table 2:** Rate of change in anthropometric characteristics, BMC (g), and BMD (g/cm^2^) for each age group according to sex.

		Male (*n* = 81)				Female (*n* = 78)			
		9–11 (*n* = 18)	12–14 (*n* = 23)	15–17 (*n* = 31)	18 ≤ (*n* = 9)	9–11 (*n* = 25)	12–14 (*n* = 19)	15–17 (*n* = 25)	18 ≤ (*n* = 9)
Weight	Percent change/year (%)	14.502 ± 2.87**	9.754 ± 5.041**	3.574 ± 2.805	2.649 ± 1.708	11.579 ± 2.960**	4.296 ± 2.548**	3.838 ± 1.775	2.734 ± 2.015
	Absolute change/year (kg)	4.323 ± 0.938**	3.595 ± 1.379**	1.873 ± 1.321	1.573 ± 1.144	3.276 ± 0.854**	1.744 ± 0.974**	1.822 ± 0.638	1.411 ± 0.942
Height	Percent change/year (%)	3.015 ± 0.653**	1.624 ± 0.740**	0.370 ± 0.500	0.310 ± 0.384	1.891 ± 0.708**	0.445 ± 0.355**	0.201 ± 0.375	0.157 ± 0.160
	Absolute change/year (cm)	4.154 ± 0.817**	2.444 ± 1.026**	0.600 ± 0.770	0.532 ± 0.651	2.555 ± 0.846**	0.672 ± 0.518**	0.292 ± 0.505	0.246 ± 0.244
BMI	Percent change/year (%)	4.574 ± 1.922	4.564 ± 2.842*	2.582 ± 1.958	1.922 ± 1.595	5.420 ± 1.992	3.053 ± 1.646*	3.287 ± 1.484	2.368 ± 2.091
	Absolute change/year (kg/m^2^)	0.708 ± 0.278	0.724 ± 0.372	0.490 ± 0.372	0.360 ± 0.358	0.812 ± 0.302	0.539 ± 0.296	0.643 ± 0.253	0.470 ± 0.348
Lumbar spine BMC	Percent change/year (%)	17.022 ± 3.979*	11.308 ± 4.154**	3.413 ± 3.530*	1.973 ± 1.947	13.974 ± 3.952*	5.069 ± 3.235**	1.310 ± 1.802*	0.937 ± 0.671
	Absolute change/year	3.833 ± 0.955**	3.339 ± 0.886**	1.396 ± 1.094**	0.968 ± 0.977	3.065 ± 0.672**	1.688 ± 0.802**	0.584 ± 0.877**	0.510 ± 0.372
Lumbar spine BMD	Percent change/year (%)	7.057 ± 1.516	5.021 ± 1.587**	1.769 ± 1.445*	0.688 ± 1.018	6.490 ± 1.691	2.588 ± 1.540**	0.944 ± 1.384*	0.711 ± 0.539
	Absolute change/year	0.038 ± 0.009	0.032 ± 0.008**	0.013 ± 0.009	0.005 ± 0.009	0.036 ± 0.007	0.018 ± 0.009**	0.008 ± 0.014	0.006 ± 0.005
Femoral neck BMC	Percent change/year (%)	6.349 ± 2.663*	4.324 ± 2.112**	0.687 ± 1.343	−0.262 ± 1.092	4.678 ± 1.967*	1.587 ± 1.395**	0.373 ± 1.152	0.264 ± 1.097
	Absolute change/year	0.182 ± 0.069**	0.140 ± 0.067**	0.025 ± 0.058	−0.014 ± 0.052	0.111 ± 0.041**	0.046 ± 0.039**	0.011 ± 0.042	0.012 ± 0.043
Femoral neck BMD	Percent change/year (%)	3.855 ± 1.899	2.425 ± 1.484	0.311 ± 1.260	−0.060 ± 1.227	3.130 ± 1.072	1.342 ± 1.241	0.531 ± 1.360	0.339 ± 1.018
	Absolute change/year	0.024 ± 0.012*	0.017 ± 0.011**	0.002 ± 0.010	−0.001 ± 0.010	0.018 ± 0.006*	0.008 ± 0.007**	0.003 ± 0.010	0.003 ± 0.008
Total hip BMC	Percent change/year (%)	12.505 ± 4.892**	7.387 ± 4.181**	1.501 ± 2.204	1.451 ± 1.496	7.420 ± 3.167**	3.151 ± 1.874**	0.893 ± 1.534	0.550 ± 1.922
	Absolute change/year	2.017 ± 0.799**	1.500 ± 0.781**	0.427 ± 0.626	0.422 ± 0.388	1.099 ± 0.359**	0.636 ± 0.366**	0.190 ± 0.381	0.151 ± 0.508
Total hip BMD	Percent change/year (%)	3.133 ± 1.633	2.654 ± 1.339**	0.620 ± 1.048	0.575 ± 1.254	2.944 ± 1.038	1.437 ± 0.921**	0.669 ± 1.513	0.246 ± 0.841
	Absolute change/year	0.023 ± 0.013	0.020 ± 0.010**	0.005 ± 0.009	0.004 ± 0.010	0.019 ± 0.007	0.010 ± 0.006**	0.005 ± 0.012	0.002 ± 0.007
Total body BMC	Percent change/year (%)	14.990 ± 1.914**	8.976 ± 2.999**	3.618 ± 2.429**	2.126 ± 1.360	9.370 ± 2.204**	4.187 ± 1.842**	1.558 ± 1.342**	0.970 ± 1.015
	Absolute change/year	148.519 ± 23.641**	118.904 ± 34.008**	62.076 ± 32.060**	42.700 ± 24.519*	91.264 ± 19.870**	53.049 ± 20.410**	24.963 ± 21.038**	17.073 ± 17.824*
Total body BMD	Percent change/year (%)	4.504 ± 1.082*	3.010 ± 0.980**	1.555 ± 0.968**	0.747 ± 0.894	3.713 ± 0.968*	1.989 ± 0.985**	0.567 ± 0.942**	0.291 ± 0.816
	Absolute change/year	0.033 ± 0.007**	0.025 ± 0.007**	0.014 ± 0.008**	0.007 ± 0.008	0.027 ± 0.006**	0.016 ± 0.007**	0.005 ± 0.008**	0.002 ± 0.008

*Note:* Statistically significant differences between males and females are given as * for *p* < 0.05 and ** for *p* < 0.01. See Supporting Information S1 for full statistical details (mean differences, 95% CIs, exact *p*‐values).

Abbreviations: BMC, bone mineral content; BMD, bone mineral density; BMI, body mass index.

The rate changes in weight and height, as well as BMC and BMD in all sites, significantly decreased with increasing age, with the highest rates observed in the age group of 9–11. In this age group, lumbar spine BMC in males showed the highest percentage of change per year with a mean of 17.02 ± 3.97 percent per year. On the other hand, total body BMC in males showed the highest absolute change per year with a mean of 148.51 ± 23.64 gr/year.

Significant differences in the weight and height change rates between males and females were observed in the baseline age groups of 9–11 and 12–14, with males exhibiting higher rates. In these two age groups, males also demonstrated significantly greater rates of change in BMC at the lumbar spine, femoral neck, total hip, and total body. Additionally, males in the 15–17 age group showed significantly higher rates of BMC change in both the lumbar spine and total body compared to females. Furthermore, during the 12–14 age group, males had notably higher rates of change in BMD at the lumbar spine, femoral neck, total hip, and total body. In the 15–17 age group, males also experienced significantly greater growth rates in TBBMD than their female counterparts.

Table 3 displays the baseline measurements of BMC and BMD at the specified sites across the five Tanner pubertal stages. The data indicate significant differences in BMC and BMD among the various Tanner groups, with an increase in these measurements from Tanner stage 1 to stage 5.

**Table 3:** Baseline BMC (g) and BMD (g/cm^2^) for each Tanner stage.

	Tanner stage					*p‐*value
	1 (*n* = 28)	2 (*n* = 22)	3 (*n* = 25)	4 (*n* = 27)	5 (*n* = 57)	
	Mean ± SD	Mean ± SD	Mean ± SD	Mean ± SD	Mean ± SD	
Lumbar spine BMC baseline	23.27 ± 5.26^a^	23.34 ± 4.03^a^	32.83 ± 6.91^b^	43.88 ± 11.90^c^	53.04 ± 10.43 ^d^	< 0.001
Lumbar spine BMD baseline	0.56 ± 0.08^a^	0.58 ± 0.05^a^	0.69 ± 0.08^b^	0.77 ± 0.13^c^	0.91 ± 0.11 ^d^	< 0.001
Femoral neck BMC baseline	2.83 ± 0.55^a^	2.65 ± 0.46^a^	3.04 ± 0.51^a^	4.06 ± 0.82^b^	4.00 ± 0.76^b^	< 0.001
Femoral neck BMD baseline	0.62 ± 0.08^a^	0.60 ± 0.08^a^	0.66 ± 0.08^a^	0.78 ± 0.11^b^	0.80 ± 0.12^b^	< 0.001
Total hip BMC baseline	16.27 ± 3.74^a^	16.32 ± 2.86^a^	21.40 ± 4.89^b^	29.89 ± 7.41^c^	29.29 ± 6.40^c^	< 0.001
Total hip BMD baseline	0.71 ± 0.08^a^	0.69 ± 0.08^a^	0.76 ± 0.09^a^	0.86 ± 0.11^b^	0.90 ± 0.11^b^	< 0.001
Total body BMC baseline	1011.89 ± 183.58^a^	1047.05 ± 145.72^a^	1282.38 ± 205.26^b^	1726.06 ± 388.92^c^	1845.59 ± 345.46^c^	< 0.001
Total body BMD baseline	0.75 ± 0.07^a^	0.76 ± 0.05^a^	0.84 ± 0.06^b^	0.92 ± 0.08^c^	0.98 ± 0.08 ^d^	< 0.001

*Note:* Means within an anatomic site (row) with the same letter are not significantly different *p* > 0.05. See Supporting Information S1 for full statistical details (mean differences, 95% CIs, exact *p*‐values).

Abbreviations: BMC, bone mineral content; BMD, bone mineral density; BMI, body mass index; SD, standard deviation.

The rates of change in BMC and BMD at each site across the different Tanner stages are demonstrated in Table 4. The growth rates are positive among all pubertal stages but most pronounced during Tanner stage 1. As participants progress through the subsequent stages of puberty, the rate of change decreases. To identify specific Tanner stages with significant differences in BMC and BMD, we employed the post hoc Tukey test. In Tables 3 and 4, means ± SDs within an anatomical site that share the same superscript letter, are not significantly different.

**Table 4:** Rate of change in BMC (g) and BMD (g/cm^2^) for each Tanner stage.

		Tanner stage					*p*‐value
		1 (*n* = 28)	2 (*n* = 22)	3 (*n* = 25)	4 (*n* = 27)	5 (*n* = 57)	
		Mean ± SD	Mean ± SD	Mean ± SD	Mean ± SD	Mean ± SD	
Lumbar spine BMC	Percent change/year (%)	16.114 ± 3.976^a^	14.359 ± 3.859^a^	7.766 ± 3.839^b^	4.686 ± 3.922^c^	1.652 ± 1.963 ^d^	< 0.001
	Absolute change/year	3.658 ± 0.843^a^	3.280 ± 0.786^a^	2.426 ± 1.080^b^	1.695 ± 1.151^b^	0.809 ± 0.926^c^	< 0.001
Lumbar spine BMD	Percent change/year (%)	6.924 ± 1.366^a^	6.269 ± 1.910^a^	3.787 ± 1.702^b^	2.237 ± 1.780^c^	1.040 ± 1.176 ^d^	< 0.001
	Absolute change/year	0.038 ± 0.007^a^	0.035 ± 0.009^a^	0.025 ± 0.010^b^	0.015 ± 0.011^c^	0.008 ± 0.011^c^	< 0.001
Femoral neck BMC	Percent change/year (%)	5.834 ± 2.434^a^	4.992 ± 2.093^a^	2.801 ± 1.935^b^	1.157 ± 1.580^c^	0.298 ± 1.168^c^	< 0.001
	Absolute change/year	0.162 ± 0.066^a^	0.130 ± 0.059^ab^	0.085 ± 0.061^b^	0.040 ± 0.063^c^	0.010 ± 0.047^c^	< 0.001
Femoral neck BMD	Percent change/year (%)	3.738 ± 1.673^a^	3.076 ± 1.138^a^	1.721 ± 1.183^b^	0.709 ± 1.293^bc^	0.344 ± 1.312^c^	< 0.001
	Absolute change/year	0.023 ± 0.011^a^	0.018 ± 0.007^ab^	0.011 ± 0.008^bc^	0.004 ± 0.010 ^cd^	0.002 ± 0.010 ^d^	< 0.001
Total hip BMC	Percent change/year (%)	10.906 ± 4.187^a^	8.596 ± 4.455^a^	4.406 ± 3.123^b^	2.656 ± 2.498^bc^	0.869 ± 1.450^c^	< 0.001
	Absolute change/year	1.726 ± 0.637^a^	1.378 ± 0.770^ab^	0.925 ± 0.735^bc^	0.690 ± 0.682^c^	0.231 ± 0.391 ^d^	< 0.001
Total hip BMD	Percent change/year (%)	3.096 ± 1.389^a^	2.988 ± 1.018^ab^	2.053 ± 1.390^b^	1.040 ± 1.139^c^	0.522 ± 1.221^c^	< 0.001
	Absolute change/year	0.022 ± 0.011^a^	0.020 ± 0.007^ab^	0.015 ± 0.010^ac^	0.008 ± 0.009 ^cd^	0.004 ± 0.010 ^d^	< 0.001
Total body BMC	Percent change/year (%)	12.732 ± 3.161^a^	10.775 ± 2.840^b^	6.087 ± 2.712^c^	4.144 ± 2.567 ^d^	1.962 ± 1.553^e^	< 0.001
	Absolute change/year	128.630 ± 37.503^a^	111.913 ± 30.353^a^	77.715 ± 36.975^b^	64.895 ± 33.839^b^	35.720 ± 2 7.888^c^	< 0.001
Total body BMD	Percent change/year (%)	4.159 ± 1.064^a^	3.916 ± 1.000^a^	2.421 ± 0.912^b^	1.635 ± 0.921^c^	0.795 ± 0.982 ^d^	< 0.001
	Absolute change/year	0.031 ± 0.007^a^	0.029 ± 0.007^a^	0.020 ± 0.007^b^	0.014 ± 0.007^b^	0.007 ± 0.009^c^	< 0.001

*Note:* Means within an anatomic site (row) with the same letter are not significantly different *p* > 0.05. See Supporting Information S1 for full statistical details (mean differences, 95% CIs, exact *p*‐values).

Abbreviations: BMC, bone mineral content; BMD, bone mineral density; BMI, body mass index; SD, standard deviation.

**Figure 1:**
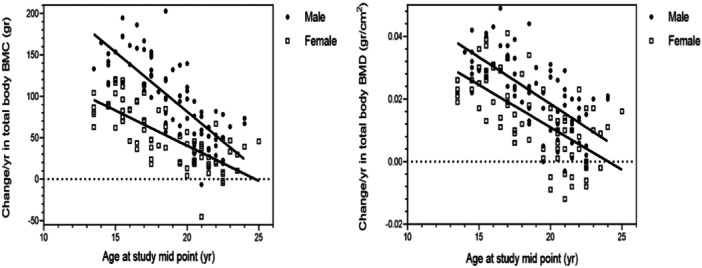
Changes in total body bone mineral content (expressed gr per year) and total body bone mineral density (expressed as gr/cm^2^ per year) by age at study mid‐point and sex. The model equations for each site and each sex were as follows: Values of change/year at each site's BMC or BMD = a (slope for each sex) * X (parameter of age at study mid‐point) + Y‐intercept for each sex.

**Figure 2:**
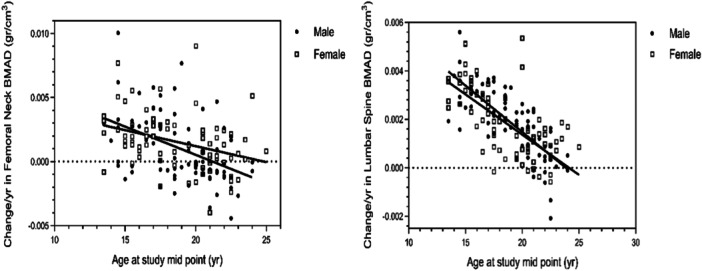
Changes in femoral neck and lumbar spine bone mineral apparent density (expressed as gr/cm^3^ per year) by age at study mid‐point and sex. The model equations for each site and each sex were as follows: Values of change/year at each site's BMAD = a (slope for each sex) * X (parameter of age at study mid‐point) + Y‐intercept for each sex.

The study found that males typically achieve peak total body BMC at an average age of 25.7 years, while females reach their peak total body BMC at 24.8 years. In terms of total body BMD, males peak at 26.2 years, whereas females reach their peak at 24 years (see Figure 1). Furthermore, the predicted ages for our participants to reach peak BMAD at femoral neck and lumbar spine were 21.2 and 23.8 years for males, and 24.8 and 24.1 years for females, respectively (see Figure 2).

Table 5 explore how age, sex, BMI, socioeconomic status index, and exercise affect total body BMC and BMD, and lumbar spine and femoral neck BMAD during adolescence and early adulthood. The findings indicate that age and BMI are the most significant factors associated with all measurements (*p* < 0.001), except for femoral neck BMAD, where age did not reach statistical significance (*p* = 0.400). Exercise also showed a significant positive correlation with all measurements: *p* < 0.001 for total body BMC and BMD, *p* = 0.035 for lumbar spine BMAD, and *p* = 0.002 for femoral neck BMAD. Additionally, male sex had a notable impact on total body BMC and BMD, with *p*‐values of < 0.001 and 0.036, respectively. In contrast, lumbar spine BMAD was significantly influenced by female sex (*p* < 0.001), while femoral neck BMAD was not affected by sex at all. Lastly, the socioeconomic status index had a minimal influence on the study measurements, showing significance only for total body BMD (*p* = 0.037).

**Table 5:** The fixed effect of age, body mass index, socioeconomic status index, gender, and exercise on total body BMC and BMD, and lumbar spine and femoral neck BMAD.

Parameter		Total body BMC			Total body BMD			Lumbar spine BMAD			Femoral neck BMAD		
		β (95% CI)	SE	*p*‐value	β (95% CI)	SE	*p*‐value	β (95% CI)	SE	*p*‐value	β (95% CI)	SE	*p*‐value
Age		57.175 (49.020 − 65.331)	4.142	< 0.001	0.015 (0.012 − 0.017)	0.001	< 0.001	0.001 (0.000 − 0.001)	0.000	< 0.001	0.000 (−0.000 − 0.000)	0.000	0.400
BMI		35.263 (26.657 − 43.870)	4.367	< 0.001	0.008 (0.006 − 0.011)	0.001	< 0.001	0.001 (0.000 − 0.001)	0.000	< 0.001	0.001 (0.001 − 0.002)	0.000	< 0.001
SES Index		7.736 (−3.618 − 19.090)	5.747	0.180	0.003 (0.000 − 0.006)	0.001	0.037	0.000 (−4.058 − 0.001)	0.000	0.066	0.000 (−0.000 − 0.001)	0.000	0.255
Gender	Male	255.099 (178.989 − 331.209)	38.561	< 0.001	0.022 (0.001 − 0.043)	0.010	0.036	—	—	—	—	—	—
	Female	—	—	—	—	—	—	0.012 (0.008 − 0.0166)	0.012	< 0.001	0.005 (−0.001 − 0.012)	0.003	0.140
Exercise	Yes	132.931 (214.294 − 51.568)	41.329	0.001	0.039 (0.062 − 0.017)	0.011	< 0.001	0.003 (0.000 − 0.006)	0.001	0.035	0.008 (0.003 − 0.013)	0.002	0.002
	No	—	—	—	—	—	—	—	—	—	—	—	—

Abbreviations: BMI, body mass index; SES, socioeconomic status.

## 4 Discussion

Osteoporosis is a disease typically affecting older adults but can be rooted in growth years. This study provides insight into the BMC, BMD, and BMAD longitudinal changes in axial skeletal sites of both male and female adolescents and young adults and determines how age, sex, puberty, BMI, socioeconomic status, and physical activity impact these bone measurements.

The study found a positive correlation between age and BMC and BMD at all measurement sites throughout the study period. As seen in the results, total body BMC and BMD increased until they plateaued and reached their peak values. However, the most significant change rates were observed in the baseline age group of 9 to 11 years old. This suggests that early adolescence is a critical window for bone development with the most rapid bone mass gain. This aligns with the results of studies indicating that 40% to 60% of adult bone mass is typically gained during early adolescence [22]. Some studies have also suggested that by age 18, around 90% of PBM has been accrued [14]. However, despite a positive correlation between age and all sites' BMC and BMD, femoral neck BMAD was not significantly associated with age. Several studies have also demonstrated that femoral neck volumetric BMD values were not influenced by age, suggesting that changes in this site are attributed primarily to increases in bone size rather than density gains [23, 24, 25]. It should be noted that BMD results could be misleading if skeletal size is not considered.

While it is widely accepted that early adolescence is the main period for bone mass accumulation, there is no consensus on the exact age at which PBM is attained. This study indicates that males and females achieve total body PBM approximately in the mid‐third decade of life. Some studies have suggested that PBM is completed during the second decade [12, 13, 26, 27]. However, evidence points to the early to mid‐third decade as the final opportunity to maximize bone mass, with several studies identifying this timeframe as when PBM is usually attained [11, 28, 29, 30, 31]. Factors contributing to discrepancies in estimated PBM ages across different studies include the specific age groups studied, racial differences, research methodologies, measurement devices used, follow‐up durations, study designs (cross‐sectional or longitudinal), and various risk factors considered in each research effort.

A significant finding of this study was the notable sex difference in BMC and BMD measurements, along with a positive correlation between male sex and total body BMC and BMD during growth years. This observation aligns with previous research indicating that males and females achieve varying levels of bone accrual due to their distinct timelines of biological maturity. Specifically, males tend to reach higher maximum levels of bone accumulation at later chronological ages compared to females [11, 26, 29]. However, despite the positive correlation between male sex and total body BMC and BMD, exceptions were noted at other skeletal sites. In our study, females exhibited higher lumbar spine BMC and BMD across all age groups, with statistically significant differences observed in specific age ranges (12–14 and 15–17 years). This phenomenon can be partially explained by the timing of growth spurts during puberty, where height growth often surpasses the mineralization process around mid‐puberty. This discrepancy can lead to a physiological decrease in lumbar and femoral BMD, resulting in increased transient incidences of fractures [10].

When adjusting for bone size by calculating BMAD, females again demonstrated a significant positive correlation with lumbar spine BMAD. This suggests that the observed sex differences in lumbar spine measurements cannot be solely attributed to variations in bone size. Several studies have documented higher lumbar spine BMD in girls compared to boys [23, 24, 27]. The literature suggests that girls accrue spinal bone mineral content earlier and at a faster rate than boys, a trend that continues until late adolescence [32]. Notably, our female participants reached their PBM at the lumbar spine several years after completing their height growth. Furthermore, although they exhibited lower rates of change in BMC and BMD across all age groups compared to males, they ultimately attained their PBM later in life. The hormonal mechanisms underlying these differences are crucial for understanding the distinct patterns of cortical and trabecular bone accrual between sexes. Androgens primarily facilitate the expansion of bone size, leading to increases in cortical bone density, while estrogen plays a vital role in mineralization at trabecular‐rich sites such as the lumbar spine [33]. These hormonal differences are essential for understanding the distinct trajectories of bone development in adolescents. Moreover, in our study, males reached their peak BMAD at the femoral neck several years earlier than females, occurring in the early third decade. Additionally, males experienced negative change rates at this site after age 18. This also suggests a sex difference in the skeletal maturation of the femoral neck; once males attain their PBM, age‐related bone loss may commence earlier compared to females at this site.

This study underscores the significant effects of puberty on BMC and BMD in both sexes, a finding that is consistent with previous studies [11, 15, 24, 25]. The results revealed substantial increases in BMC and BMD between Tanner stages 2 and 3, as well as between stages 3 and 4. These findings suggest that mid‐puberty is a critical phase for bone development, characterized by elevated levels of growth hormone and sex steroids. Research conducted by Sabatier et al. corroborates these findings, indicating that up to 60% of total bone mass can be accrued across all skeletal sites during the transition from Tanner stages 2 to 4 [34].

The study found that BMI has a positive relationship with total body BMC and BMD, and BMAD in the lumbar spine and femoral neck. This relationship is consistent with previous research showing a significant association of BMI with these measures during adolescence and early adulthood [35, 36, 37]. Additionally, a meta‐analysis and systematic review discovered that overweight or obese individuals have higher BMD than those with a healthy weight [38]. Even though obesity positively affects BMD, several studies have indicated that having a high BMI significantly increases the risk of developing morbid conditions. The underlying mechanisms include oxidative stress, inflammation, and mitochondrial dysfunction [39, 40, 41]. Faienza et al. suggested that these obesity mechanisms can increase the risk of osteoporosis and fractures [42]. Therefore, adolescents must maintain an appropriate BMI to strike a balance between obesity and bone mass.

The study's findings suggested that exercise positively impacts bone acquisition during adolescence and early adulthood. Exercise is a more specific form of physical activity involving planned, organized, and repetitive movements to maintain or improve physical fitness and health outcomes, such as bone strength [43]. Lu et al. found no evidence of habitual physical activity affecting total body BMC and BMD from childhood into young adulthood [29]. Nevertheless, literature has demonstrated that participation in high levels of physical activity is linked to greater bone mass accrual compared to less active peers. Level A evidence supports the beneficial impact of physical activity and exercise on BMC and BMD [6]. Physical activity is beneficial for enhancing bone mineral mass accumulation in children and adolescents, with a stronger impact before rather than during or after pubertal maturation [44]. Further research is needed to investigate how different types of mechanical loading, such as exercise magnitude and frequency, affect bone mass and geometry during growth [45].

Another study result was the association of socioeconomic status with total body BMD in adolescence and early adulthood. Socioeconomic status was shown to be linked with various acute and chronic diseases, including osteoporosis [46, 47]. Additionally, it was documented that elderly individuals with low socioeconomic status are at a higher risk of hip fracture [48, 49]. However, results regarding the association between socioeconomic status and BMD are inconsistent. The study by Garcia‐Marco did not find evidence of the association between socioeconomic status indicators and bone mass [50]. In contrast, Arabi et al. found a correlation between lower socioeconomic status and lower BMD [24]. Also, it has been shown that having a higher socioeconomic status in childhood is linked to stronger bones at the femoral neck and higher lumbar BMD in adulthood [51, 52]. Thus, further longitudinal studies are necessary to examine the relationship between socioeconomic status parameters and bone mass, aiming to identify potential targets for intervention.

In addition to the determinants of the present research, a number of comorbidities and prevailing environmental conditions may play a role in the heterogeneity of bone mineral accretion during adolescence and young adulthood. Eating disorders and food intolerances, for example, may have a deleterious effect on nutrient intake and the regulation of hormones and therefore retard bone development [53, 54]. Similarly, type 1 diabetes has been reported to be strongly associated with decreased BMD and impaired bone quality in children and teenagers, with emphasis on the role of long‐term metabolic illness in skeletal integrity [55]. Even more recently, the COVID‐19 pandemic has also emerged as a causative element in the outcomes of bone health, with physical activity restriction, shifts in dietary habits, and increased psychosocial stress being shown to negatively affect BMD in children and adolescents [56, 57]. While these factors were beyond the focus of the present study, their incorporation in future longitudinal studies can provide an even more comprehensive picture in relation to the determinants of optimal bone mass as well as the interindividual variation in bone health patterns.

When interpreting our results, it is important to consider both the strengths and limitations of our study. One significant strength is its longitudinal design, which allows for a more accurate estimation of the age at which peak bone mass is achieved and the factors influencing this process, unlike cross‐sectional studies. We also considered various risk factors, including pubertal stage, socioeconomic status, and physical activity, all of which play a crucial role in determining PBM. To assess bone mineral content in grams and bone mineral density in grams per square centimeter, we utilized dual‐energy x‐ray absorptiometry despite its inherent limitations. DXA is a two‐dimensional imaging technique that calculates BMD by dividing BMC by the projected bone area. However, this approach does not accurately reflect true volumetric density, as it does not take into account bone depth. To address this limitation, we incorporated bone mineral apparent density, which is proposed to reduce size‐related biases in BMD results and estimate volumetric bone density in grams per cubic centimeter [20]. Further consideration is that our study's design included only two DXA assessments per participant, spaced approximately ten years apart. While this long interval provides valuable longitudinal perspective, it does not capture shorter‐term fluctuations in bone accrual. However, this methodological approach is consistent with previous longitudinal studies of bone mineral accrual [9] and provides meaningful insight into long‐term trends and the timing of peak bone mass.

## 5 Conclusion

According to this study's findings, we concluded that the rates of BMC and BMD increase were higher during adolescence than in early adulthood, during which the rate of changes declined. The acquisition of PBM occurred in the mid‐third decade of life, with females reaching their peaks earlier than males. Older age, higher BMI, higher socioeconomic status, male sex, and exercise positively affected total body BMC and BMD during adolescence and early adulthood. This study identified the crucial years during which skeletal mass accumulates and factors affecting bone mass, which can aid in developing intervention protocols and planning for achieving maximal PBM to prevent the development of osteoporosis later in life.

## Author Contributions

**Arzhang Naseri:** investigation, methodology, resources, software, project administration, supervision, validation, visualization, writing – original draft. **Farnaz Atighi:** methodology, software, validation, visualization, writing – review and editing. **Alireza Keshtkar:** methodology, software, validation, visualization, writing – review and editing. **Pedram Talezadeh:** funding acquisition, resources, validation, visualization. **Marzieh Bakhshayeshkaram:** investigation, resources, supervision, validation. **Marjan Jeddi:** conceptualization, supervision, validation. **Mohammad Mahdi Dabbaghmanesh:** investigation, resources, validation, visualization. **Seyed Taghi Heydari:** data curation, formal analysis, methodology, software, validation. **Naeimehossadat Asmarian:** data curation, formal analysis, methodology, software; validation. **Mohammad Hossein Dabbaghmanesh:** conceptualization, funding acquisition, methodology, project administration, resources, supervision, validation.

## Conflicts of Interest

The authors declare no conflicts of interest.

## Transparency Statement

The lead author Mohammad Hossein Dabbaghmanesh affirms that this article is an honest, accurate, and transparent account of the study being reported; that no important aspects of the study have been omitted; and that any discrepancies from the study as planned (and, if relevant, registered) have been explained.

## Supporting information

Supporting Material.

## Data Availability

The data that support the findings of this study are available from the corresponding author upon reasonable request. The corresponding author had full access to all of the data in this study and takes complete responsibility for the integrity of the data and the accuracy of the data analysis.
