# Supplementary material for: Bone Mineral Accrual From Adolescence Into Young Adulthood and Peak Bone Mass: A Longitudinal Cohort Study
Source: Health Sci Rep. 2025 Oct 28;8(11):e71411. doi: 10.1002/hsr2.71411 (PMC12560013; doi:10.1002/hsr2.71411)
Supplement: Supplementary file 1 — Supporting Material. [file HSR2-8-e71411-s001.docx]

Table 1- Mean Differences, 95% Confidence Intervals, and p-values for Baseline Anthropometric Characteristics, BMC (g), and BMD (g/cm²) Across Age Groups by Sex

| Variable | Age Group | Mean Difference | 95% CI Lower | 95% CI Upper | p-value |
| --- | --- | --- | --- | --- | --- |
| Weight (kg) | 9-11 | 1.50 | -1.59 | 4.58 | 0.334 |
|  | 12-14 | -1.35 | -7.29 | 4.60 | 0.650 |
|  | 15-17 | 6.18 | -0.16 | 12.52 | 0.056 |
|  | 18-19 | 8.11 | -2.58 | 18.81 | 0.127 |
| Height (cm) | 9-11 | 0.91 | -3.96 | 5.78 | 0.708 |
|  | 12-14 | 1.80 | -3.99 | 7.59 | 0.533 |
|  | 15-17 | 12.70 | 8.31 | 17.10 | <0.001 |
|  | 18-19 | 17.33 | 10.30 | 24.37 | <0.001 |
| BMI (kg/m²) | 9-11 | 0.58 | -0.50 | 1.67 | 0.283 |
|  | 12-14 | -1.16 | -2.91 | 0.60 | 0.190 |
|  | 15-17 | -0.73 | -2.83 | 1.38 | 0.492 |
|  | 18-19 | -1.33 | -4.49 | 1.82 | 0.383 |
| Lumbar Spine BMC | 9-11 | -0.08 | -2.58 | 2.42 | 0.947 |
|  | 12-14 | -3.73 | -8.84 | 1.37 | 0.147 |
|  | 15-17 | -0.04 | -6.40 | 6.33 | 0.991 |
|  | 18-19 | 2.17 | -8.52 | 12.87 | 0.672 |
| Lumbar Spine BMD | 9-11 | -0.031 | -0.074 | 0.012 | 0.155 |
|  | 12-14 | -0.070 | -0.131 | -0.009 | 0.025 |
|  | 15-17 | -0.091 | -0.159 | -0.022 | 0.011 |
|  | 18-19 | -0.061 | -0.199 | 0.077 | 0.364 |
| Femoral Neck BMC | 9-11 | 0.45 | 0.19 | 0.71 | >0.001 |
|  | 12-14 | 0.38 | 0.03 | 0.73 | 0.037 |
|  | 15-17 | 0.83 | 0.46 | 1.19 | <0.001 |
|  | 18-19 | 0.96 | 0.32 | 1.61 | 0.006 |
| Femoral Neck BMD | 9-11 | 0.051 | 0.008 | 0.094 | 0.020 |
|  | 12-14 | 0.073 | 0.018 | 0.128 | 0.011 |
|  | 15-17 | 0.053 | -0.007 | 0.113 | 0.084 |
|  | 18-19 | 0.054 | -0.085 | 0.193 | 0.420 |
| Total Hip BMC | 9-11 | 0.90 | -0.85 | 2.64 | 0.305 |
|  | 12-14 | 2.28 | -1.20 | 5.77 | 0.193 |
|  | 15-17 | 7.98 | 5.12 | 10.84 | <0.001 |
|  | 18-19 | 6.94 | 0.87 | 13.01 | 0.028 |
| Total Hip BMD | 9-11 | 0.053 | 0.007 | 0.099 | 0.026 |
|  | 12-14 | 0.037 | -0.022 | 0.097 | 0.215 |
|  | 15-17 | 0.047 | -0.011 | 0.105 | 0.113 |
|  | 18-19 | 0.020 | -0.115 | 0.155 | 0.759 |
| Total Body BMC | 9-11 | 8.89 | -62.13 | 79.92 | 0.802 |
|  | 12-14 | 88.19 | -79.18 | 255.56 | 0.293 |
|  | 15-17 | 182.13 | 2.16 | 362.11 | 0.047 |
|  | 18-19 | 285.13 | -79.30 | 649.55 | 0.117 |
| Total Body BMD | 9-11 | 0.013 | -0.022 | 0.049 | 0.460 |
|  | 12-14 | 0.030 | -0.013 | 0.073 | 0.161 |
|  | 15-17 | -0.009 | -0.056 | 0.039 | 0.718 |
|  | 18-19 | 0.009 | -0.090 | 0.107 | 0.856 |

Table 2- Mean Differences, 95% Confidence Intervals, and p-values for Rates of Change in Anthropometric Characteristics, BMC (g), and BMD (g/cm²) Across Age Groups by Sex

| Variable | | Age Group | Mean Difference | 95% CI Lower | 95% CI Upper | p-value |
| --- | --- | --- | --- | --- | --- | --- |
| Weight | Percent change/year (%) | 9-11 | 2.92 | 1.06 | 4.78 | 0.003 |
|  |  | 12-14 | 5.46 | 2.88 | 8.03 | <0.001 |
|  |  | 15-17 | -0.26 | -1.56 | 1.03 | 0.684 |
|  |  | 18-19 | -0.08 | -2.01 | 1.84 | 0.927 |
|  | Absolute change/year (kg) | 9-11 | 1.05 | 0.48 | 1.61 | 0.001 |
|  |  | 12-14 | 1.85 | 1.09 | 2.61 | <0.001 |
|  |  | 15-17 | 0.05 | -0.53 | 0.63 | 0.860 |
|  |  | 18-19 | 0.16 | -0.93 | 1.25 | 0.757 |
| Height | Percent change/year (%) | 9-11 | 1.12 | 0.69 | 1.56 | <0.001 |
|  |  | 12-14 | 1.18 | 0.80 | 1.55 | <0.001 |
|  |  | 15-17 | 0.17 | -0.07 | 0.41 | 0.168 |
|  |  | 18-19 | 0.15 | -0.16 | 0.47 | 0.312 |
|  | Absolute change/year (cm) | 9-11 | 1.60 | 1.07 | 2.13 | <0.001 |
|  |  | 12-14 | 1.77 | 1.25 | 2.30 | <0.001 |
|  |  | 15-17 | 0.31 | -0.05 | 0.67 | 0.090 |
|  |  | 18-19 | 0.29 | -0.24 | 0.81 | 0.262 |
| BMI | Percent change/year (%) | 9-11 | -0.85 | -2.09 | 0.40 | 0.179 |
|  |  | 12-14 | 1.51 | 0.02 | 3.00 | 0.047 |
|  |  | 15-17 | -0.71 | -1.66 | 0.24 | 0.142 |
|  |  | 18-19 | -0.45 | -2.36 | 1.46 | 0.626 |
|  | Absolute change/year (kg/m^2^) | 9-11 | -0.10 | -0.29 | 0.08 | 0.265 |
|  |  | 12-14 | 0.18 | -0.03 | 0.40 | 0.087 |
|  |  | 15-17 | -0.15 | -0.33 | 0.02 | 0.087 |
|  |  | 18-19 | -0.11 | -0.48 | 0.26 | 0.533 |
| Lumbar spine BMC | Percent change/year (%) | 9-11 | 3.05 | 0.52 | 5.57 | 0.019 |
|  |  | 12-14 | 6.24 | 3.88 | 8.60 | <0.001 |
|  |  | 15-17 | 2.10 | 0.53 | 3.67 | 0.010 |
|  |  | 18-19 | 1.04 | -0.42 | 2.49 | 0.151 |
|  | Absolute change/year | 9-11 | 0.77 | 0.25 | 1.28 | 0.004 |
|  |  | 12-14 | 1.65 | 1.12 | 2.18 | <0.001 |
|  |  | 15-17 | 0.81 | 0.26 | 1.36 | 0.004 |
|  |  | 18-19 | 0.46 | -0.28 | 1.20 | 0.208 |
| Lumbar spine BMD | Percent change/year (%) | 9-11 | 0.57 | -0.46 | 1.60 | 0.272 |
|  |  | 12-14 | 2.43 | 1.45 | 3.41 | <0.001 |
|  |  | 15-17 | 0.83 | 0.05 | 1.60 | 0.038 |
|  |  | 18-19 | -0.02 | -0.84 | 0.79 | 0.952 |
|  | Absolute change/year | 9-11 | 0.002 | -0.004 | 0.007 | 0.513 |
|  |  | 12-14 | 0.014 | 0.009 | 0.020 | <0.001 |
|  |  | 15-17 | 0.005 | -0.001 | 0.012 | 0.119 |
|  |  | 18-19 | -0.002 | -0.009 | 0.006 | 0.677 |
| Femoral neck BMC | Percent change/year (%) | 9-11 | 1.67 | 0.25 | 3.10 | 0.023 |
|  |  | 12-14 | 2.74 | 1.59 | 3.88 | <0.001 |
|  |  | 15-17 | 0.31 | -0.38 | 1.00 | 0.366 |
|  |  | 18-19 | -0.53 | -1.62 | 0.57 | 0.323 |
|  | Absolute change/year | 9-11 | 0.07 | 0.04 | 0.10 | <0.001 |
|  |  | 12-14 | 0.09 | 0.06 | 0.13 | <0.001 |
|  |  | 15-17 | 0.01 | -0.01 | 0.04 | 0.316 |
|  |  | 18-19 | -0.03 | -0.07 | 0.02 | 0.252 |
| Femoral neck BMD | Percent change/year (%) | 9-11 | 0.73 | -0.19 | 1.64 | 0.119 |
|  |  | 12-14 | 1.08 | 0.22 | 1.95 | 0.015 |
|  |  | 15-17 | -0.22 | -0.93 | 0.49 | 0.538 |
|  |  | 18-19 | -0.40 | -1.53 | 0.73 | 0.462 |
|  | Absolute change/year | 9-11 | 0.006 | 0.001 | 0.012 | 0.033 |
|  |  | 12-14 | 0.009 | 0.003 | 0.015 | 0.007 |
|  |  | 15-17 | -0.002 | -0.007 | 0.004 | 0.538 |
|  |  | 18-19 | -0.005 | -0.014 | 0.005 | 0.306 |
| Total Hip BMC | Percent change/year (%) | 9-11 | 5.09 | 2.60 | 7.57 | <0.001 |
|  |  | 12-14 | 4.24 | 2.14 | 6.33 | <0.001 |
|  |  | 15-17 | 0.61 | -0.45 | 1.67 | 0.255 |
|  |  | 18-19 | 0.90 | -0.82 | 2.62 | 0.283 |
|  | Absolute change/year | 9-11 | 0.92 | 0.55 | 1.28 | <0.001 |
|  |  | 12-14 | 0.86 | 0.47 | 1.26 | <0.001 |
|  |  | 15-17 | 0.24 | -0.06 | 0.53 | 0.110 |
|  |  | 18-19 | 0.27 | -0.18 | 0.72 | 0.223 |
| Total Hip BMD | Percent change/year (%) | 9-11 | 0.19 | -0.63 | 1.01 | 0.645 |
|  |  | 12-14 | 1.22 | 0.48 | 1.95 | 0.002 |
|  |  | 15-17 | -0.05 | -0.74 | 0.64 | 0.888 |
|  |  | 18-19 | 0.33 | -0.75 | 1.41 | 0.523 |
|  | Absolute change/year | 9-11 | 0.003 | -0.003 | 0.010 | 0.302 |
|  |  | 12-14 | 0.010 | 0.004 | 0.016 | 0.001 |
|  |  | 15-17 | 0.000 | -0.006 | 0.006 | 1.000 |
|  |  | 18-19 | 0.002 | -0.008 | 0.011 | 0.723 |
| Total Body BMC | Percent change/year (%) | 9-11 | 5.62 | 4.32 | 6.92 | <0.001 |
|  |  | 12-14 | 4.79 | 3.19 | 6.38 | <0.001 |
|  |  | 15-17 | 2.06 | 0.95 | 3.17 | <0.001 |
|  |  | 18-19 | 1.16 | -0.04 | 2.36 | 0.058 |
|  | Absolute change/year | 9-11 | 57.26 | 43.82 | 70.69 | <0.001 |
|  |  | 12-14 | 65.85 | 47.87 | 83.84 | <0.001 |
|  |  | 15-17 | 37.11 | 21.94 | 52.28 | <0.001 |
|  |  | 18-19 | 25.63 | 4.21 | 47.05 | 0.022 |
| Total Body BMD | Percent change/year (%) | 9-11 | 0.79 | 0.16 | 1.43 | 0.016 |
|  |  | 12-14 | 1.02 | 0.41 | 1.64 | 0.002 |
|  |  | 15-17 | 0.99 | 0.47 | 1.50 | <0.001 |
|  |  | 18-19 | 0.46 | -0.40 | 1.31 | 0.276 |
|  | Absolute change/year | 9-11 | 0.006 | 0.002 | 0.011 | 0.005 |
|  |  | 12-14 | 0.009 | 0.004 | 0.014 | <0.001 |
|  |  | 15-17 | 0.009 | 0.005 | 0.014 | <0.001 |
|  |  | 18-19 | 0.004 | -0.004 | 0.013 | 0.281 |

Table 3- Mean Differences, 95% Confidence Intervals, and p-values for Baseline BMC (g) and BMD (g/cm²) Across Tanner Stages

| Bone Parameter | Tanner Comparison | Mean Difference | 95% CI Lower | 95% CI Upper | p-value |
| --- | --- | --- | --- | --- | --- |
| Lumbar Spine BMC baseline | 1 vs 2 | -0.07 | -7.18 | 7.04 | >0.99 |
|  | 1 vs 3 | -9.56 | -16.34 | -2.77 | 0.001 |
|  | 1 vs 4 | -20.61 | -27.33 | -13.89 | <0.001 |
|  | 1 vs 5 | -29.76 | -35.47 | -24.05 | <0.001 |
|  | 2 vs 3 | -9.49 | -16.72 | -2.25 | 0.004 |
|  | 2 vs 4 | -20.54 | -27.71 | -13.37 | <0.001 |
|  | 2 vs 5 | -29.70 | -35.94 | -23.46 | <0.001 |
|  | 3 vs 4 | -11.05 | -17.90 | -4.21 | <0.001 |
|  | 3 vs 5 | -20.21 | -26.07 | -14.35 | <0.001 |
|  | 4 vs 5 | -9.15 | -14.94 | -3.37 | <0.001 |
| Lumbar Spine BMD baseline | 1 vs 2 | -0.02 | -0.10 | 0.07 | 0.969 |
|  | 1 vs 3 | -0.13 | -0.21 | -0.05 | <0.001 |
|  | 1 vs 4 | -0.22 | -0.30 | -0.13 | <0.001 |
|  | 1 vs 5 | -0.35 | -0.42 | -0.28 | <0.001 |
|  | 2 vs 3 | -0.11 | -0.20 | -0.02 | 0.005 |
|  | 2 vs 4 | -0.20 | -0.28 | -0.11 | <0.001 |
|  | 2 vs 5 | -0.33 | -0.41 | -0.26 | <0.001 |
|  | 3 vs 4 | -0.08 | -0.17 | -0.00 | 0.043 |
|  | 3 vs 5 | -0.22 | -0.29 | -0.15 | <0.001 |
|  | 4 vs 5 | -0.14 | -0.21 | -0.07 | <0.001 |
| Femoral Neck BMC baseline | 1 vs 2 | 0.18 | -0.35 | 0.71 | 0.883 |
|  | 1 vs 3 | -0.22 | -0.73 | 0.29 | 0.767 |
|  | 1 vs 4 | -1.23 | -1.73 | -0.73 | <0.001 |
|  | 1 vs 5 | -1.17 | -1.60 | -0.74 | <0.001 |
|  | 2 vs 3 | -0.39 | -0.94 | 0.15 | 0.263 |
|  | 2 vs 4 | -1.41 | -1.94 | -0.88 | <0.001 |
|  | 2 vs 5 | -1.35 | -1.82 | -0.88 | <0.001 |
|  | 3 vs 4 | -1.02 | -1.53 | -0.50 | <0.001 |
|  | 3 vs 5 | -0.96 | -1.40 | -0.51 | <0.001 |
|  | 4 vs 5 | 0.06 | -0.37 | 0.49 | 0.995 |
| Femoral Neck BMD baseline | 1 vs 2 | 0.02 | -0.06 | 0.10 | 0.962 |
|  | 1 vs 3 | -0.04 | -0.12 | 0.04 | 0.620 |
|  | 1 vs 4 | -0.16 | -0.24 | -0.08 | <0.001 |
|  | 1 vs 5 | -0.18 | -0.24 | -0.11 | <0.001 |
|  | 2 vs 3 | -0.06 | -0.15 | 0.02 | 0.278 |
|  | 2 vs 4 | -0.18 | -0.26 | -0.09 | <0.001 |
|  | 2 vs 5 | -0.20 | -0.27 | -0.12 | <0.001 |
|  | 3 vs 4 | -0.12 | -0.20 | -0.04 | 0.001 |
|  | 3 vs 5 | -0.13 | -0.20 | -0.07 | <0.001 |
|  | 4 vs 5 | -0.02 | -0.09 | 0.05 | 0.944 |
| Total Hip BMC baseline | 1 vs 2 | -0.04 | -4.45 | 4.37 | >0.99 |
|  | 1 vs 3 | -5.13 | -9.39 | -0.87 | 0.010 |
|  | 1 vs 4 | -13.61 | -17.79 | -9.44 | <0.001 |
|  | 1 vs 5 | -13.02 | -16.60 | -9.44 | <0.001 |
|  | 2 vs 3 | -5.08 | -9.61 | -0.56 | 0.019 |
|  | 2 vs 4 | -13.57 | -18.02 | -9.12 | <0.001 |
|  | 2 vs 5 | -12.98 | -16.87 | -9.08 | <0.001 |
|  | 3 vs 4 | -8.49 | -12.78 | -4.19 | <0.001 |
|  | 3 vs 5 | -7.89 | -11.62 | -4.17 | <0.001 |
|  | 4 vs 5 | 0.59 | -3.03 | 4.22 | 0.991 |
| Total Hip BMD baseline | 1 vs 2 | 0.01 | -0.07 | 0.10 | 0.987 |
|  | 1 vs 3 | -0.05 | -0.13 | 0.03 | 0.405 |
|  | 1 vs 4 | -0.15 | -0.23 | -0.08 | <0.001 |
|  | 1 vs 5 | -0.19 | -0.26 | -0.13 | <0.001 |
|  | 2 vs 3 | -0.07 | -0.15 | 0.02 | 0.208 |
|  | 2 vs 4 | -0.17 | -0.25 | -0.09 | <0.001 |
|  | 2 vs 5 | -0.21 | -0.28 | -0.14 | <0.001 |
|  | 3 vs 4 | -0.10 | -0.18 | -0.02 | 0.004 |
|  | 3 vs 5 | -0.14 | -0.21 | -0.07 | <0.001 |
|  | 4 vs 5 | -0.04 | -0.11 | 0.03 | 0.487 |
| Total Body BMC baseline | 1 vs 2 | -35.15 | -263.50 | 193.19 | 0.993 |
|  | 1 vs 3 | -270.49 | -491.03 | -49.95 | 0.008 |
|  | 1 vs 4 | -714.17 | -930.35 | -497.99 | <0.001 |
|  | 1 vs 5 | -833.70 | -1018.66 | -648.73 | <0.001 |
|  | 2 vs 3 | -235.33 | -469.63 | -1.03 | 0.048 |
|  | 2 vs 4 | -679.01 | -909.21 | -448.82 | <0.001 |
|  | 2 vs 5 | -798.54 | -999.71 | -597.37 | <0.001 |
|  | 3 vs 4 | -443.68 | -666.14 | -221.23 | <0.001 |
|  | 3 vs 5 | -563.21 | -755.47 | -370.95 | <0.001 |
|  | 4 vs 5 | -119.53 | -306.78 | 67.72 | 0.400 |
| Total Body BMD baseline | 1 vs 2 | -0.01 | -0.07 | 0.05 | 0.991 |
|  | 1 vs 3 | -0.09 | -0.15 | -0.03 | 0.001 |
|  | 1 vs 4 | -0.17 | -0.23 | -0.11 | <0.001 |
|  | 1 vs 5 | -0.23 | -0.28 | -0.18 | <0.001 |
|  | 2 vs 3 | -0.08 | -0.14 | -0.01 | 0.009 |
|  | 2 vs 4 | -0.16 | -0.22 | -0.10 | <0.001 |
|  | 2 vs 5 | -0.22 | -0.28 | -0.17 | <0.001 |
|  | 3 vs 4 | -0.08 | -0.14 | -0.02 | 0.002 |
|  | 3 vs 5 | -0.14 | -0.20 | -0.09 | <0.001 |
|  | 4 vs 5 | -0.06 | -0.11 | -0.01 | 0.010 |

Table 4- Mean Differences, 95% Confidence Intervals, and p-values for Rates of Change in BMC (g) and BMD (g/cm²) Across Tanner Stages

| Dependent Variable | | | | | Tanner Comparison | Mean Difference | 95% CI Lower | 95% CI Upper | p-value |
| --- | --- | --- | --- | --- | --- | --- | --- | --- | --- |
| Lumbar spine BMC | | | | Percent change/year (%) | 1 vs 2 | 1.76 | -0.91 | 4.42 | 0.366 |
|  |  |  |  |  | 1 vs 3 | 8.35 | 5.81 | 10.89 | <0.001 |
|  |  |  |  |  | 1 vs 4 | 11.43 | 8.89 | 13.97 | <0.001 |
|  |  |  |  |  | 1 vs 5 | 14.46 | 12.32 | 16.60 | <0.001 |
|  |  |  |  |  | 2 vs 3 | 6.59 | 3.88 | 9.30 | <0.001 |
|  |  |  |  |  | 2 vs 4 | 9.67 | 6.96 | 12.38 | <0.001 |
|  |  |  |  |  | 2 vs 5 | 12.71 | 10.37 | 15.04 | <0.001 |
|  |  |  |  |  | 3 vs 4 | 3.08 | 0.49 | 5.67 | 0.011 |
|  |  |  |  |  | 3 vs 5 | 6.11 | 3.92 | 8.31 | <0.001 |
|  |  |  |  |  | 4 vs 5 | 3.03 | 0.84 | 5.23 | 0.002 |
|  |  |  |  | Absolute change/year | 1 vs 2 | 0.38 | -0.40 | 1.15 | 0.661 |
|  |  |  |  |  | 1 vs 3 | 1.23 | 0.49 | 1.97 | <0.001 |
|  |  |  |  |  | 1 vs 4 | 1.96 | 1.23 | 2.70 | <0.001 |
|  |  |  |  |  | 1 vs 5 | 2.85 | 2.23 | 3.47 | <0.001 |
|  |  |  |  |  | 2 vs 3 | 0.85 | 0.07 | 1.64 | 0.026 |
|  |  |  |  |  | 2 vs 4 | 1.59 | 0.80 | 2.37 | <0.001 |
|  |  |  |  |  | 2 vs 5 | 2.47 | 1.79 | 3.15 | <0.001 |
|  |  |  |  |  | 3 vs 4 | 0.73 | -0.02 | 1.48 | 0.061 |
|  |  |  |  |  | 3 vs 5 | 1.62 | 0.98 | 2.25 | <0.001 |
|  |  |  |  |  | 4 vs 5 | 0.89 | 0.25 | 1.52 | 0.002 |
| Lumbar Spine BMD | | | | Percent change/year (%) | 1 vs 2 | 0.65 | -0.56 | 1.87 | 0.574 |
|  |  |  |  |  | 1 vs 3 | 3.14 | 1.97 | 4.30 | <0.001 |
|  |  |  |  |  | 1 vs 4 | 4.69 | 3.53 | 5.85 | <0.001 |
|  |  |  |  |  | 1 vs 5 | 5.88 | 4.91 | 6.86 | <0.001 |
|  |  |  |  |  | 2 vs 3 | 2.48 | 1.24 | 3.72 | <0.001 |
|  |  |  |  |  | 2 vs 4 | 4.03 | 2.79 | 5.27 | <0.001 |
|  |  |  |  |  | 2 vs 5 | 5.23 | 4.16 | 6.30 | <0.001 |
|  |  |  |  |  | 3 vs 4 | 1.55 | 0.37 | 2.73 | 0.004 |
|  |  |  |  |  | 3 vs 5 | 2.75 | 1.74 | 3.75 | <0.001 |
|  |  |  |  |  | 4 vs 5 | 1.20 | 0.19 | 2.20 | 0.011 |
|  |  |  |  | Absolute change/year | 1 vs 2 | 0.003 | -0.006 | 0.011 | 0.902 |
|  |  |  |  |  | 1 vs 3 | 0.013 | 0.005 | 0.021 | <0.001 |
|  |  |  |  |  | 1 vs 4 | 0.023 | 0.015 | 0.031 | <0.001 |
|  |  |  |  |  | 1 vs 5 | 0.030 | 0.023 | 0.036 | <0.001 |
|  |  |  |  |  | 2 vs 3 | 0.010 | 0.002 | 0.019 | 0.008 |
|  |  |  |  |  | 2 vs 4 | 0.020 | 0.012 | 0.029 | <0.001 |
|  |  |  |  |  | 2 vs 5 | 0.027 | 0.020 | 0.034 | <0.001 |
|  |  |  |  |  | 3 vs 4 | 0.010 | 0.002 | 0.018 | 0.007 |
|  |  |  |  |  | 3 vs 5 | 0.017 | 0.010 | 0.023 | <0.001 |
|  |  |  |  |  | 4 vs 5 | 0.007 | -0.000 | 0.014 | 0.053 |
| Femoral Neck BMC | | | Percent change/year (%) | | 1 vs 2 | 0.84 | -0.55 | 2.24 | 0.459 |
|  |  |  |  |  | 1 vs 3 | 3.03 | 1.68 | 4.38 | <0.001 |
|  |  |  |  |  | 1 vs 4 | 4.68 | 3.36 | 6.00 | <0.001 |
|  |  |  |  |  | 1 vs 5 | 5.54 | 4.40 | 6.67 | <0.001 |
|  |  |  |  |  | 2 vs 3 | 2.19 | 0.76 | 3.62 | <0.001 |
|  |  |  |  |  | 2 vs 4 | 3.83 | 2.43 | 5.24 | <0.001 |
|  |  |  |  |  | 2 vs 5 | 4.69 | 3.46 | 5.93 | <0.001 |
|  |  |  |  |  | 3 vs 4 | 1.64 | 0.28 | 3.00 | 0.009 |
|  |  |  |  |  | 3 vs 5 | 2.50 | 1.33 | 3.68 | <0.001 |
|  |  |  |  |  | 4 vs 5 | 0.86 | -0.29 | 2.01 | 0.240 |
|  |  |  | Absolute change/year | | 1 vs 2 | 0.032 | -0.014 | 0.078 | 0.304 |
|  |  |  |  |  | 1 vs 3 | 0.077 | 0.033 | 0.121 | <0.001 |
|  |  |  |  |  | 1 vs 4 | 0.122 | 0.079 | 0.165 | <0.001 |
|  |  |  |  |  | 1 vs 5 | 0.152 | 0.115 | 0.189 | <0.001 |
|  |  |  |  |  | 2 vs 3 | 0.045 | -0.002 | 0.092 | 0.065 |
|  |  |  |  |  | 2 vs 4 | 0.090 | 0.044 | 0.136 | <0.001 |
|  |  |  |  |  | 2 vs 5 | 0.120 | 0.080 | 0.160 | <0.001 |
|  |  |  |  |  | 3 vs 4 | 0.045 | 0.001 | 0.090 | 0.045 |
|  |  |  |  |  | 3 vs 5 | 0.075 | 0.036 | 0.113 | <0.001 |
|  |  |  |  |  | 4 vs 5 | 0.030 | -0.008 | 0.067 | 0.188 |
| Femoral Neck BMD | | | Percent change/year (%) | | 1 vs 2 | 0.66 | -0.39 | 1.72 | 0.416 |
|  |  |  |  |  | 1 vs 3 | 2.02 | 1.00 | 3.03 | <0.001 |
|  |  |  |  |  | 1 vs 4 | 3.03 | 2.03 | 4.03 | <0.001 |
|  |  |  |  |  | 1 vs 5 | 3.39 | 2.54 | 4.25 | <0.001 |
|  |  |  |  |  | 2 vs 3 | 1.35 | 0.27 | 2.44 | 0.006 |
|  |  |  |  |  | 2 vs 4 | 2.37 | 1.30 | 3.43 | <0.001 |
|  |  |  |  |  | 2 vs 5 | 2.73 | 1.80 | 3.66 | <0.001 |
|  |  |  |  |  | 3 vs 4 | 1.01 | -0.01 | 2.04 | 0.055 |
|  |  |  |  |  | 3 vs 5 | 1.38 | 0.49 | 2.27 | <0.001 |
|  |  |  |  |  | 4 vs 5 | 0.37 | -0.50 | 1.23 | 0.772 |
|  |  |  | Absolute change/year | | 1 vs 2 | 0.005 | -0.003 | 0.013 | 0.399 |
|  |  |  |  |  | 1 vs 3 | 0.012 | 0.005 | 0.020 | <0.001 |
|  |  |  |  |  | 1 vs 4 | 0.019 | 0.011 | 0.026 | <0.001 |
|  |  |  |  |  | 1 vs 5 | 0.021 | 0.015 | 0.028 | <0.001 |
|  |  |  |  |  | 2 vs 3 | 0.007 | -0.001 | 0.015 | 0.109 |
|  |  |  |  |  | 2 vs 4 | 0.014 | 0.006 | 0.022 | <0.001 |
|  |  |  |  |  | 2 vs 5 | 0.016 | 0.009 | 0.023 | <0.001 |
|  |  |  |  |  | 3 vs 4 | 0.007 | -0.001 | 0.014 | 0.112 |
|  |  |  |  |  | 3 vs 5 | 0.009 | 0.002 | 0.016 | 0.002 |
|  |  |  |  |  | 4 vs 5 | 0.002 | -0.004 | 0.009 | 0.873 |
| Total Hip BMC | | Percent change/year (%) | | | 1 vs 2 | 2.31 | -0.07 | 4.69 | 0.062 |
|  |  |  |  |  | 1 vs 3 | 6.45 | 4.15 | 8.75 | <0.001 |
|  |  |  |  |  | 1 vs 4 | 8.25 | 6.00 | 10.50 | <0.001 |
|  |  |  |  |  | 1 vs 5 | 10.04 | 8.10 | 11.97 | <0.001 |
|  |  |  |  |  | 2 vs 3 | 4.14 | 1.69 | 6.58 | <0.001 |
|  |  |  |  |  | 2 vs 4 | 5.94 | 3.54 | 8.34 | <0.001 |
|  |  |  |  |  | 2 vs 5 | 7.73 | 5.62 | 9.83 | <0.001 |
|  |  |  |  |  | 3 vs 4 | 1.80 | -0.52 | 4.12 | 0.206 |
|  |  |  |  |  | 3 vs 5 | 3.59 | 1.58 | 5.60 | <0.001 |
|  |  |  |  |  | 4 vs 5 | 1.79 | -0.17 | 3.75 | 0.092 |
|  |  | Absolute change/year | | | 1 vs 2 | 0.35 | -0.13 | 0.83 | 0.270 |
|  |  |  |  |  | 1 vs 3 | 0.80 | 0.34 | 1.26 | <0.001 |
|  |  |  |  |  | 1 vs 4 | 1.04 | 0.58 | 1.49 | <0.001 |
|  |  |  |  |  | 1 vs 5 | 1.50 | 1.11 | 1.89 | <0.001 |
|  |  |  |  |  | 2 vs 3 | 0.45 | -0.04 | 0.94 | 0.088 |
|  |  |  |  |  | 2 vs 4 | 0.69 | 0.20 | 1.17 | 0.001 |
|  |  |  |  |  | 2 vs 5 | 1.15 | 0.72 | 1.57 | <0.001 |
|  |  |  |  |  | 3 vs 4 | 0.24 | -0.23 | 0.70 | 0.634 |
|  |  |  |  |  | 3 vs 5 | 0.69 | 0.29 | 1.10 | <0.001 |
|  |  |  |  |  | 4 vs 5 | 0.46 | 0.06 | 0.85 | 0.014 |
| Total Hip BMD | | Percent change/year (%) | | | 1 vs 2 | 0.11 | -0.87 | 1.09 | 0.998 |
|  |  |  |  |  | 1 vs 3 | 1.04 | 0.10 | 1.99 | 0.022 |
|  |  |  |  |  | 1 vs 4 | 2.06 | 1.13 | 2.98 | <0.001 |
|  |  |  |  |  | 1 vs 5 | 2.57 | 1.78 | 3.37 | <0.001 |
|  |  |  |  |  | 2 vs 3 | 0.93 | -0.07 | 1.94 | 0.081 |
|  |  |  |  |  | 2 vs 4 | 1.95 | 0.96 | 2.93 | <0.001 |
|  |  |  |  |  | 2 vs 5 | 2.47 | 1.60 | 3.33 | <0.001 |
|  |  |  |  |  | 3 vs 4 | 1.01 | 0.06 | 1.96 | 0.031 |
|  |  |  |  |  | 3 vs 5 | 1.53 | 0.71 | 2.36 | <0.001 |
|  |  |  |  |  | 4 vs 5 | 0.52 | -0.28 | 1.32 | 0.388 |
|  |  | Absolute change/year | | | 1 vs 2 | 0.001 | -0.007 | 0.009 | 0.987 |
|  |  |  |  |  | 1 vs 3 | 0.007 | -0.001 | 0.014 | 0.113 |
|  |  |  |  |  | 1 vs 4 | 0.014 | 0.006 | 0.021 | <0.001 |
|  |  |  |  |  | 1 vs 5 | 0.018 | 0.012 | 0.024 | <0.001 |
|  |  |  |  |  | 2 vs 3 | 0.005 | -0.003 | 0.013 | 0.378 |
|  |  |  |  |  | 2 vs 4 | 0.012 | 0.004 | 0.020 | <0.001 |
|  |  |  |  |  | 2 vs 5 | 0.017 | 0.010 | 0.024 | <0.001 |
|  |  |  |  |  | 3 vs 4 | 0.007 | -0.001 | 0.015 | 0.090 |
|  |  |  |  |  | 3 vs 5 | 0.011 | 0.004 | 0.018 | <0.001 |
|  |  |  |  |  | 4 vs 5 | 0.004 | -0.002 | 0.011 | 0.424 |
| Total Body BMC | Percent change/year (%) | | | | 1 vs 2 | 1.96 | 0.03 | 3.89 | 0.045 |
|  |  |  |  |  | 1 vs 3 | 6.64 | 4.78 | 8.51 | <0.001 |
|  |  |  |  |  | 1 vs 4 | 8.59 | 6.76 | 10.41 | <0.001 |
|  |  |  |  |  | 1 vs 5 | 10.77 | 9.20 | 12.34 | <0.001 |
|  |  |  |  |  | 2 vs 3 | 4.69 | 2.71 | 6.67 | <0.001 |
|  |  |  |  |  | 2 vs 4 | 6.63 | 4.69 | 8.58 | <0.001 |
|  |  |  |  |  | 2 vs 5 | 8.81 | 7.11 | 10.52 | <0.001 |
|  |  |  |  |  | 3 vs 4 | 1.94 | 0.06 | 3.82 | 0.039 |
|  |  |  |  |  | 3 vs 5 | 4.12 | 2.50 | 5.75 | <0.001 |
|  |  |  |  |  | 4 vs 5 | 2.18 | 0.60 | 3.77 | 0.002 |
|  | Absolute change/year | | | | 1 vs 2 | 16.72 | -8.93 | 42.36 | 0.378 |
|  |  |  |  |  | 1 vs 3 | 50.91 | 26.14 | 75.68 | <0.001 |
|  |  |  |  |  | 1 vs 4 | 63.74 | 39.45 | 88.02 | <0.001 |
|  |  |  |  |  | 1 vs 5 | 92.91 | 72.07 | 113.74 | <0.001 |
|  |  |  |  |  | 2 vs 3 | 34.20 | 7.88 | 60.51 | 0.004 |
|  |  |  |  |  | 2 vs 4 | 47.02 | 21.16 | 72.87 | <0.001 |
|  |  |  |  |  | 2 vs 5 | 76.19 | 53.54 | 98.84 | <0.001 |
|  |  |  |  |  | 3 vs 4 | 12.82 | -12.16 | 37.81 | 0.618 |
|  |  |  |  |  | 3 vs 5 | 42.00 | 20.34 | 63.65 | <0.001 |
|  |  |  |  |  | 4 vs 5 | 29.17 | 8.08 | 50.27 | 0.002 |
| Total Body BMD | Percent change/year (%) | | | | 1 vs 2 | 0.24 | -0.53 | 1.01 | 0.907 |
|  |  |  |  |  | 1 vs 3 | 1.74 | 0.99 | 2.48 | <0.001 |
|  |  |  |  |  | 1 vs 4 | 2.52 | 1.80 | 3.25 | <0.001 |
|  |  |  |  |  | 1 vs 5 | 3.36 | 2.74 | 3.99 | <0.001 |
|  |  |  |  |  | 2 vs 3 | 1.50 | 0.70 | 2.29 | <0.001 |
|  |  |  |  |  | 2 vs 4 | 2.28 | 1.51 | 3.06 | <0.001 |
|  |  |  |  |  | 2 vs 5 | 3.12 | 2.44 | 3.80 | <0.001 |
|  |  |  |  |  | 3 vs 4 | 0.79 | 0.04 | 1.54 | 0.035 |
|  |  |  |  |  | 3 vs 5 | 1.63 | 0.98 | 2.27 | <0.001 |
|  |  |  |  |  | 4 vs 5 | 0.84 | 0.21 | 1.47 | 0.003 |
|  | Absolute change/year | | | | 1 vs 2 | 0.002 | -0.005 | 0.008 | 0.968 |
|  |  |  |  |  | 1 vs 3 | 0.011 | 0.005 | 0.017 | <0.001 |
|  |  |  |  |  | 1 vs 4 | 0.017 | 0.010 | 0.023 | <0.001 |
|  |  |  |  |  | 1 vs 5 | 0.024 | 0.018 | 0.029 | <0.001 |
|  |  |  |  |  | 2 vs 3 | 0.009 | 0.003 | 0.016 | 0.002 |
|  |  |  |  |  | 2 vs 4 | 0.015 | 0.008 | 0.022 | <0.001 |
|  |  |  |  |  | 2 vs 5 | 0.022 | 0.016 | 0.028 | <0.001 |
|  |  |  |  |  | 3 vs 4 | 0.006 | -0.001 | 0.012 | 0.116 |
|  |  |  |  |  | 3 vs 5 | 0.013 | 0.007 | 0.018 | <0.001 |
|  |  |  |  |  | 4 vs 5 | 0.007 | 0.002 | 0.012 | 0.005 |
